# A case study on the effect of light and colors in the built environment on autistic children’s behavior

**DOI:** 10.3389/fpsyt.2022.1042641

**Published:** 2022-11-30

**Authors:** Ashwini Sunil Nair, Radhakrishnan Shanthi Priya, Prashanthini Rajagopal, Chandramouli Pradeepa, Ramalingam Senthil, Samiappan Dhanalakshmi, Khin Wee Lai, Xiang Wu, Xiaowei Zuo

**Affiliations:** ^1^School of Architecture and Interior Design, SRM Institute of Science and Technology, Chennai, India; ^2^Department of Mechanical Engineering, SRM Institute of Science and Technology, Chennai, India; ^3^Department of Electronics and Communication Engineering, SRM Institute of Science and Technology, Chennai, India; ^4^Department of Biomedical Engineering, Faculty of Engineering, Universiti Malaya, Kuala Lumpur, Malaysia; ^5^School of Medical Information and Engineering, Xuzhou Medical University, Xuzhou, China; ^6^Department of Psychiatry, The Affiliated Xuzhou Oriental Hospital of Xuzhou Medical University, Xuzhou, Jiangsu, China

**Keywords:** autistic children, cognitive functioning, wall colors, indoor lighting, built environment, behavioral changes

## Abstract

**Background:**

The importance of strategies and services by caregivers and family members substantially impact the psychological and emotional wellbeing of autistic children. The rapid research developments in clinical and non-clinical methods benefit the features of autistic children. Among various internal and external factors, the influence of the built environment also impacts the characteristics of autistic children. This study investigates primarily the psychological effect of light and colors on the mood and behavior of autistic children to identify the most favorable and preferred indoor lights and color shades.

**Methods:**

A questionnaire survey was conducted at an autism center among autistic children and their parents. This study included autistic children aged between 6 and 16 (45 males, 42 females, mean age 8.7 years, standard deviation 2.3). Eighty-seven participants were involved in the survey to determine the sensory perceptions, intolerance, preferences, and sensitivities of children with an autism spectrum disorder toward colors and lighting. The margin of error at the statistical analysis’s 95% confidence level is ± 0.481.

**Results:**

As per this case report, the children have various color preferences and respond differently to different shades. Different hues have varying effects on autistic children, with many neutral tones and mellow shades proven to be autistic-friendly with their calming and soothing effect, while bright, bold, and intense colors are refreshing and stimulating. The stimulus of bright-lighting causes behavioral changes in autistic children prone to light sensitivity.

**Conclusion:**

The insights gained from this interaction with parents and caretakers of autistic children could be helpful for designers to incorporate specific autistic-friendly design elements that make productive interior spaces. A complete understanding of the effect of factors like color and lighting on the learning ability and engagement of autistic children in an indoor environment is essential for designers and clinicians. The main findings of this study could be helpful for a designer and clinicians to address designing an autism-friendly built environment with a color palette and lighting scheme conducive to their wellbeing and to maximize their cognitive functioning.

## Introduction

A significant instance of pervasive development being witnessed throughout the globe is the complex neurodevelopment condition called autism spectrum disorder (ASD) which impacts behavior, communication, and social interaction (1–4). The scientific community has conducted several research studies in recent years to assess the incidence and prevalence of ASD (5–7). A significant increase in ASD could be globally recognized (8–10). India is a highly populated nation of about 1.3 billion families with children under 15, representing almost many inhabitants per a detailed clinical evaluation (11, 12). More than two million individuals in India have been impacted by autism. This challenging observation has led to user-centric strategies, support and intervention services such as healthcare facilities, education support, and rehabilitation services to facilitate easy interaction and seamless social integration. The need to cater to the unique needs, preferences, requirements and challenges of autistic children has directed the efforts of designers toward considering these essential aspects while designing inclusive and empowering spaces for children on the autism spectrum (13, 14). ASD is characterized by cognition, understanding, social behavior, and emotional expression. People with autism have challenges deciphering emotional expressions, cannot comprehend the emotions of others, avoid eye contact, and have extreme sensitivity to the environment. Such sights or sounds could influence a positive change and behavior (15). Research studies have shown that the ASD group demonstrated greater perceptual and learning characteristics (16–18). Challenging features of executive functioning are commonly observed in people experiencing ASD, with noticeable suppression of cognitive flexibility; such behavioral inflexibility impacts their ability to perform any task (19).

Autistic children tend to perceive and experience their environment differently from others (20, 21). By paying attention to the sensory sensitivities and challenges with visual information processing faced by autistic children, appropriate changes can be incorporated into their indoor environment to turn it into a friendly and accommodative place tailored to their unique needs. Suggestions were made optional but solicited to understand the user’s needs before designing an inclusive space for autistic children. This calming environment would encourage their growth and facilitate their learning regarding the visual environment and other influencing factors. The survey method provided valuable insights into the preliminary considerations for designing an engaging and welcoming indoor environment that provides positive sensory experiences and alleviates anxiety.

Any autism-friendly design depends first and foremost on the end-user’s unique needs, preferences, and comfort. The design considerations for sensory-friendly spaces must be based on the core aspects of functionality, connectivity and responsiveness following a careful study of the inherent features of the built environment and after gaining a good understanding of the unique perspectives of autistic individuals (22). Design mechanisms must also thoroughly examine essential factors such as imagination, verbal/non-verbal communication, social interaction, sensory issues, behavior, and safety. There continue to be several research studies on the many facets of autism, resulting in various scientific advancements and noteworthy developments. However, evidence shows that many factors remain unknown. Cognitive deficits and motor coordination challenges are common in children with autism. They have a detrimental impact on children’s everyday lives and limit their achievements. Due to the increased effort in caring for autistic children and finding ways to cope with their behavioral challenges, parents should be familiar with the environmental impacts (23, 24). Autistic children tend to have a dysfunctional sensory system; this sensory processing disorder is perceived as the biggest challenge to surmount (25, 26). Few but well-chosen articles and publications provide valuable insights into all aspects of one vital concept: the senses.

Information comes through all the senses, which are processed and organized by the brain. Processing sensory information is extremely difficult for autistic children, affecting their sensory responsiveness and reactions. Human senses are interconnected and cannot be separated, but multisensory integration is challenging for autistic children. The eight sensory systems are visual, auditory, olfactory, gustatory, tactile, proprioceptive, vestibular, and interoception. These are highly central to understanding sensory processing in autism and a sensory integration approach would make them very relevant to investigating visual processing (27–29). Sensory processing disorder is often a comorbid symptom of ASD, but not all children with sensory processing disorder have autism.

According to the literature, sensory difficulties and processing disorders appear to be more prevalent in autistic children and ASD is perceived to be the root cause of their sensory system (30). People with sensory processing disorders are generally classified as hypo and hypersensitive (under and over-responsiveness). The response and reaction of both categories to low and high stimuli in any setting tend to differ based on how they process sensory information. Individuals with autism are frequently more socially aware due to sensory difficulties with seeing, hearing, and feeling. Children with autism do not interact much with others, struggle with sensory overload, have difficulty expressing their feelings, and prefer to live in their private world of self-imposed isolation. Despite social, behavioral and communication challenges and sensory processing challenges, they have a strong understanding of their surroundings, have exceptional memories, are very creative and possess the ability to learn new skills (31). Thus, sensory integration issues can continually make the built environment unpleasant and hostile for many people on the autism spectrum.

Since ASD impairments can fluctuate between moderate and severe with few or mild characteristics and with co-occurring conditions, the abilities, needs and services demand to vary wildly across individuals. However, almost all require therapeutic interventions and support for their lifespan (32). Apart from a functional link with human health, emotions influence individuals’ interactions with environmental factors. In the long run, the features arising from misguided attempts to “regularize” the perception of an autistic individual may prompt corrective behavioral responses such as repeated motions and other methods to regain some predictability in sensory inputs. Free and open communication is essential because it affects how one understands, investigates, and evaluates surroundings (33). The emotional responses that arise throughout the relationship between a human and the different ecosystem entities might align with the earlier statement (34). Much clinical evidence is available in the Indian setting on ASD. The active involvement and participation of parents of children and young adults in intervention, integration, and rehabilitation efforts help in gaining a better understanding of autistic behavior, which is essential for all productive experimentation with the goal of better developmental, behavioral, and educational outcomes (35).

Many researchers have investigated the relationship between the five senses and their impact on the surrounding environment with multiple parameters. Investigating the quality and dynamics of early encounters is a difficult task. It usually necessitates observing and integrating multimodal social signals and comprehending how two interactions synchronize for understanding the autistic child’s behavior (36, 37). Such understanding can significantly help designers design a supportive, autism-friendly environment and help them innovate in the design considerations of various inclusive spaces. Researchers have experimented, analyzed and briefly explained specific design considerations, elements and components for users on the autistic spectrum. The following design components help to understand the importance and need for a unique, autistic-friendly built environment for preliminary studies.

### Space organization

The spatial element is a crucial factor that designers and architects must be aware of when building for people with ASD. This is attributed to the importance of routine, order, regularity, and predictability. Well-defined areas should be placed in a logical sequence based on the regular schedule followed in specific settings regarding spatial sequencing to make it easier for autistic children to navigate these spaces without assistance. A research study was conducted to recommend the setting up of an organized space focusing on accessibility to and inclusivity of autistic children by creating surroundings that were clearly defined, orderly, simple, safe, satisfying, predictable, welcoming, and stable (38–40). Spatial experiences are a method of using spatial knowledge to decode the arrangement of built spaces. A research study provided an overview of the Faison School in the United States. It demonstrated how adaptable and customizable layouts with sensory benefits could benefit autistic children with a sensory processing disorder. Introducing clearly defined strips along corridors is a component of spatial sequence and navigation aids that make indoor spaces reachable, inviting, and comfortable for autistics. The color of those strips is also crucial in the case of spatial navigation and when wayfinding techniques are deployed.

### Wayfinding

This technique develops a simple and easy navigational approach by incorporating assistive visual aids such as landmarks, decoration, and color-coding (32). Environmental navigation, orientation, and wayfinding were explored in a research study conducted in a school for autistic children (34). Before the study, the researcher spent time in the participants’ classrooms to become better acquainted with the users’ everyday routines and observe their behaviors and interactions during class activities, which helped frame their actions during wayfinding tasks (41, 42). Various metrics were employed in evidence-based research to examine the effects of spatial navigational aids and the setting up of demarcated sensory-friendly zones such as sensory rooms. Children with sensory sensitivities could use it as a safe place to withdraw or shelter when overwhelmed by a sensory overload. A simple navigation system can help visually sensitive autistic users traverse their environment quickly and without fear. Color-coded paths can help them easily navigate from one location to another (43).

### Lighting

The importance of good lighting, both natural and artificial, is evident in the way it can transform any space. Lighting has a significant impact on the sensory system of autistic children. Evidence-based research was conducted on an active group of autistic users to determine their responses to lighting, their light sensitivity and possible light modifications that could be implemented for people with sensory challenges (44, 45). The diffusive light effects on the walls were thoroughly studied through windows, floors, ceilings, and furniture. The study analyzed how the users reacted to light in the hallway when the change in hue caused corresponding changes in users’ behavior and mood. The study also explored adjustable lighting settings to match the natural circadian cycle of the body closely. Indirect lighting reduces flickering, intensity, and brightness, helping ASD individuals cope with their light sensitivity (38). Artificial lights should ideally be fitted with dimming controls to alter or produce a luminous interior as an indirect light source (13). Before the research observation of the study group of autistic children began each morning, a checklist was implemented to validate the accurate establishment of the route and to verify if the circumstances between the participants were consistent, remarkably everyday sensory stimuli like light and sound. To verify if circumstances between the participants are consistent, particularly considering light and sound, potentially observe autistic children.

Based on the environmental conditions, the responses gathered included the children’s reactions to light (amount of illumination) and sound (number of decibels), which were studied to devise ways to help autistic children struggling with sound and light sensitivity. Ideally, the environmental circumstances should be constant for all participants, especially since bright lights and loud sounds can be bothersome sensory overloads to autistic children affecting their functioning and behavior. The restriction of visual stimulation was enforced through dimmer switches provided for all lighting installations to reduce light levels based on the need (46). Students could easily regulate the degree and intensity of light stimulation by switching each light row as needed.

### Acoustics

Sound quality is perhaps the most critical component in managing and maintaining autism spectrum conduct on an even keel among all the visual inputs involved in building design (47). Therefore, attempts must be made to reduce the auditory sensitivity of autistic children and prevent sensory overload. An experiential study showed that lowering sound levels and echoes in zones packed with autistic students resulted in improved mental attentiveness, service quality, a better quality of work, and reduced the tempo of compulsive self-stimulating behavior (32). The authors indicated that due to the intolerance of extraneous noise and difficulties in auditory processing, auditory sensitivity measures while planning interior acoustics are essential for building environments housing people with autism. Therefore, architects must avoid excessive ambient noise and effectively manage the acoustic requirements of enclosed spaces to prevent the extra stimulation from a noisy space that could distress autistic children with sound sensitivity in a space.

By identifying and eliminating interfering sensory information under the user’s needs and creating a quiet and comfortable environment conducive to productivity, people with autism can enhance their attention, reduce stress, and prevent inappropriate behavior. The authors advised the integration of pink cacophony for privacy and a salutogenic sound design approach in a space with many activities. A lower sound intensity of less than 50 dB is preferable for autistic children and beyond 60 dB makes it inappropriate. It was also recommended that a suitable acoustic environment within an enclosed space by reducing the frequency of the room’s resonance for acoustic comfort and soundproofing a room by ensuring good sound absorption.

### Colors

Autistic children are sensitive to colors depending on how they perceive them. Most see them with greater intensity than they are. Colors in interior spaces affect their mood, learning, and behavior and must be chosen judiciously (42, 43). Colorful mat boards were chosen in a research study to make color shapes on the floor as the lightweight material was not a potential hazard. It was ensured that their color complimented the aids placed by the door to establish a uniform color pattern. The colored indicators, colored doors, and floor forms enabled traceability and consistency. The study’s findings revealed that colorful signs made areas more accessible and easier to navigate for autistic children. Using autism-friendly color palettes such as pastel shades, neutral colors, and muted tones can foster a soothing sensory experience in an indoor environment. Bold and bright colors must be avoided as they could be over-stimulating and disturbing, which may cause autistic children to become tense and aggressive. Autistic children keenly feel different from others due to a lack of self-confidence and an inability to adapt. The importance of using color therapy to assist and empower autistic children to function without discomfort in a calm environment and in correcting their behavioral abnormalities must be recognized (48).

Investigations are necessary to examine the impact of color indices on learning specific complex tasks. Thus, the variable effect of indices on generalization and persistence is mainly in the case of color formation. Investigations are necessary to examine the psychological impact and effects of color on learning specific complex tasks and cognitive task performance by autistic children. Evidently, with the understanding of the existing scenario and the available data, it seems worth examining the application of color techniques for autistic individuals (49). Using organic material and neutral and relaxing color tones is deemed suitable for an autistic educational environment as they are conducive to learning, increase attention and boost energy levels. Psychological features are practical with selective colors. Red or yellow can be problematic as they can agitate, depress or confuse autistic children causing their withdrawal (13). An uncommon experimental study was conducted to determine if concepts could be taught to autistic children by associating them with color (50). The authors attempted to explain an inclination model simply by associating colors with symmetrical items objects (51–53).

People pick colors linked to things they like and do not prefer the colors they dislike (54). Autistic children generally have atypical color preferences and aversions with complex emotional associations with color (55, 56). Their visual perceptions of color can influence their emotions and behavior (57–60). High contrast choices by participants with ASD may be due to color compulsions and may be attributed to a preference for items in colors they like. A study of participants with ASD found that color preferences and obsessions could be linked to a preferred object, resulting in higher color preference than usual (50).

### Safety

Protecting autistic children from harm by providing a safe and secure environment is vital as their sensory processing ability is not developed enough to recognize challenges and handle crises (13). As children with autism often prefer to flee and escape frightening situations, it is necessary to establish standard procedures or safety systems that make it impossible for them to leave any place unnoticed places or amenities. Generally, any place’s structure, organization, and design must be ensured that it offers maximum freedom and flexibility, eliminates all problematic situations, addresses safety concerns, and is tailored to the behavioral factors unique to ASD users. Although all possible chances cannot be eliminated, they must be anticipated; safety issues must be duly considered, and safety strategies must be customized, as ASD children are susceptible to many challenges. Integrating primary entry points of safety systems enables the monitoring and tracking of the movement of people. For example, establishing exterior barriers improves safety aspects and facilities better organization, particularly in open spaces and courts. Installing mirrors with rounded corners in restrooms, using reflectors and fixing wider toilets are simple and easy measures that can be adopted to make areas safe, comfortable, easily accessible, and autism-friendly zones.

In most research studies, the preferences and opinions of autistic people are kept for analysis at a later stage. Several studies have highlighted the importance of clear rules, consistent routines, and calm orderliness for the adaptive functioning of individuals with ASD. Such studies are to help them cope with a predictable environment, situation, or setting without unexpected changes and help them to be engaged without being overwhelmed by anxiety (61, 62). As they face language difficulties and communication challenges, schedules and structure are very important as they provide much-needed stability and help improve their wellbeing.

Many behavioral changes are attributed to different colors, and many research studies have attempted to decode the physiological effects of colors observed in autistic people. A study found that the strategic use of color could foster learning and suggested that the materials for learning activities could be chosen based on favorable color perceptions (63). The fixtures and furnishings provided in an enclosed space also play a crucial role in the sensory experiences of an autistic person while transitioning from one space to another. Loud and noisy disturbances are usually not tolerated by autistic individuals as they have auditory sensitivity. These jarring stimulants can serve as triggers and bring about behavior changes ranging from mild to severe. Surveys on autistic users have provided an exceptional understanding of how they perceive space and spatial relations.

Many diverse research studies conducted by various scholars have extensively investigated the fundamental aspects relevant to enabling an autism-friendly environment (50). However, the core concepts of color, light and texture, all fundamental design aspects, must be linked to the essential components of visual and tactile sensory learning. These interconnected factors can provide an enriching experience for autistic individuals when they are integrated. Not many quantitative research studies have explored creating an independent, sensory-friendly environment for autistic individuals. The initial findings of some research studies have indicated that design elements such as color, light, and texture must be studied collectively. Determining the combined influence of color, light, and texture on interior spaces makes them more accommodating for autistic people with sensory intolerance. The perception of psychological changes, emotional expressions, and behavioral alterations in autistic children could be studied further to enhance their features.

Although several diseases fall under the gambit of ASD, this study focuses on autistic children’s visual sensitivity. The understanding of the sensory perceptions of ordinary people is evident. Understanding the sensory perceptions of ASD people are challenging to determine their human psychology toward colors and lights. The novelty of the present study is a non-clinical approach to identifying the favorable visual environment for ASD children using interaction with the parents and caretakers of children with ASD. The indoor domestic environment has been critically investigated to determine if it is conducive to the unique needs and requirements of the children. Many environmental circumstances can affect the behavior, learning, performance, functioning, and wellbeing of autistic children, such as sound, smell, temperature, sense perceptions, communication, and social interactions. This investigative study is limited to the influence and impact of the two crucial sensory factors of light and color on autistic children in addition to space, wayfinding, and acoustics. This case study is primarily aimed at improving the quality of life of children with ASD through architectural aspects of the indoor built environment.

The present study examines the sensory impact of light and color in an indoor environment on autistic children whose senses are generally more heightened than usual. The insights on sensory triggers gained from this study can guide designers and clinicians while designing living spaces and taking steps to reduce stimuli that may lead to behavioral features. Understanding ASD is difficult due to neurological abnormality and a complex behaviorally defined development disorder with multiple contributing factors. With the increasing prevalence of autism worldwide, the need for progressive architectural design standards, guidelines and best practices to improve the built environment, explicitly incorporating sensory sensitivity strategies in interior spaces that cater to autistic requirements, is being keenly felt. There is greater recognition for design’s impact in providing a soothing yet engaging environment for children with special needs. The primary objective is to share the fundamental factors to be considered in an indoor space and the tools and techniques that can be deployed to transform it into a calm, supportive, and autism-friendly zone, thereby resolving several adaptive issues for autistic children.

The primary objectives of the present study are as follows:

• Identify the central impairments of sensory intolerance in autism and understand the functional impact of light and color on autistic children.
• To provide a pleasant sensory experience of light and color to make them feel safe and secure within the built environment.
• Investigate the perception of light and color by autistic children and their influence through a comprehensive survey with various parameters.
• The current work is summarized in the following sections. **Section 1** provides the background and needs of the present study. **Section 2** elaborates on the methods adopted in this case study. **Section 3** discusses the results of the case study. **Section 4** describes the discussion of the results. **Section 5** summarizes the significant conclusions and further scope of the research.

## Materials and methods

### Data collection

**FIGURE 1:**
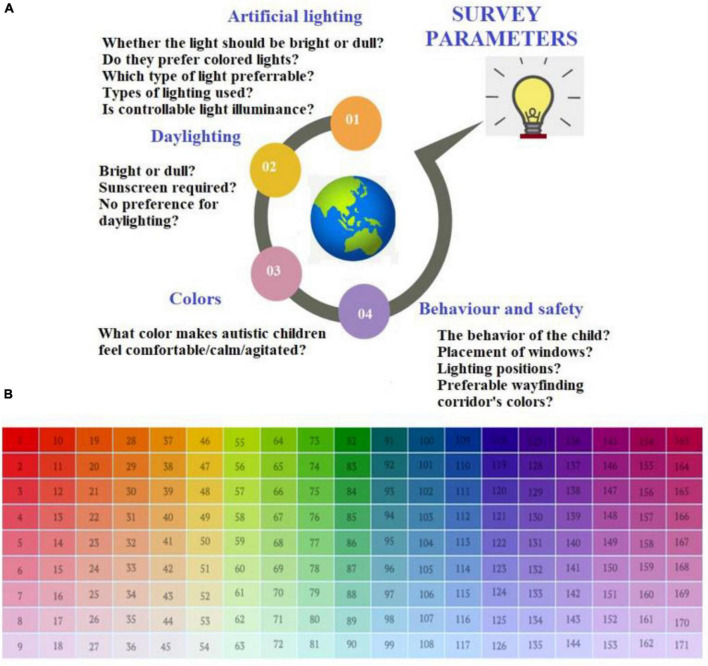
**(A)** Parameters used for framing the questionnaire of the present study, **(B)** color matrix prepared to understand user preferences and behavior.

The research commenced with an in-depth literature review carried out in an organized manner to identify the current knowledge and understanding of ASD before framing the questionnaire contents (64–66). The questionnaire survey for the quantitative analysis consisted of forty questions that comprehensively covered the primary design aspects that need to be considered, in terms of light and color perceptions, before designing interior spaces intended for autistic children. The questionnaire was carefully designed in a lucid and straightforward format from the literature (67–73). The questionnaire was fine-tuned further in consultation with the therapists. The questionnaire survey was followed by ranking to indicate the category of the factor under consideration: low (Not at all suitable–one-point scale, suitable–two-point scale)–moderate (neutral–three-point scale), high (Most suitable), not necessarily essential. All the research questions in the questionnaire had this scale as a ranking guide. Figure 1A shows the various parameters used for the questionnaire of the present study. Figure 1B illustrates the color matrix used to understand user preferences and behavior.

The questionnaire survey was conducted with the parents of autistic children in-person mode. Informed consent was obtained from the participants before the interaction. The data was collected manually from Autism Schools in Northern India. Responses were collected from parents and caretakers of autistic children by conducting a structured interview per the prepared questionnaire. The data collected was then statistically analyzed and interpreted the results. The survey was first conducted with eighty-seven participants, all parents, and caretakers of children with autism.

Prior to commencing the interview, parents were briefed about the purpose of the research study, its envisaged contribution and its importance. The parents and caretakers were notified about the survey’s objectives and informed of the importance of the questionnaire survey. The main intention of the questionnaire was to understand the sensory preferences of autistic children in an indoor environment regarding light and color factors.

The questionnaire was divided into three sections, details of which are as follows.

• Section A comprises the names of the parents or the therapist/psychologist, the child’s age, gender and city of residence.
• Section B includes open-ended questions about artificial and daylight conditions, window position, lighting position, the color of lights, degree of brightness, light color temperature, and favorable behavioral changes that can be brought about by altering lighting conditions.
• Section C presents a color matrix. The child or the parent picks up the color of their choice based on parameters for gauging the children’s mood, whether they feel comfortable, uncomfortable, calm, and patient, or disturbed. Colors are chosen for different rooms and preferences are noted to determine autism-friendly colors. Section C also comprises generic questions regarding preferences for sensory dimensions of space and the importance of visual stimuli in providing favorable sensory experiences.

To gain an understanding of the behavior of autistic children, the survey was conducted based on Section A, comprising of age, gender, and city of residence of the study participants. For this survey, eighty-seven participants were considered, amongst which 45 were male and 42 were female. Section B comprises a comprehensive questionnaire to determine the impact of light on autistic children. Table 1 shows the statistical analysis of the selected participants.

**TABLE 1:** The statistical analysis of the selected population of 87 participants.

Parameter	Values
Arithmetic mean and geometric mean	8.7 and 8.4
Median and mode	9 (both)
Range and count	10 and 87
Smallest and largest	6 and 16
Variance	5.25
Standard deviation	2.29
The margin of error at a 95% confidence level	±0.481

Appendix A provides insights into the section comprising three parts where the focus group is mainly children falling into the age group of 6–16 years, with the overall count being eighty-seven participants. The survey was conducted at autism centers under a professional practitioner’s guidance.

## Results

The analysis and the questionnaire survey findings are presented in this section with sub-sections for light and color, respectively. The standard deviation of the survey method is 2.3. The margin of error and coefficient of variation at a 95% confidence level is ± 0.85 and 0.27, respectively. This confirms that the results are within the permissible values.

### Analysis of light

**FIGURE 2:**
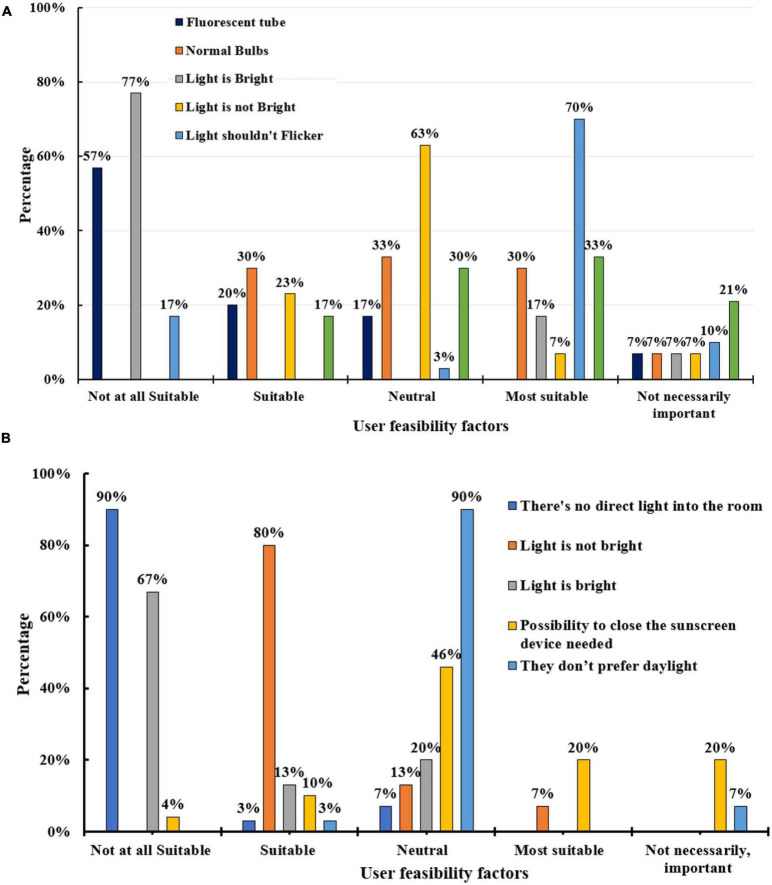
**(A)** Artificial lighting conditions, **(B)** natural light conditions.

**FIGURE 3:**
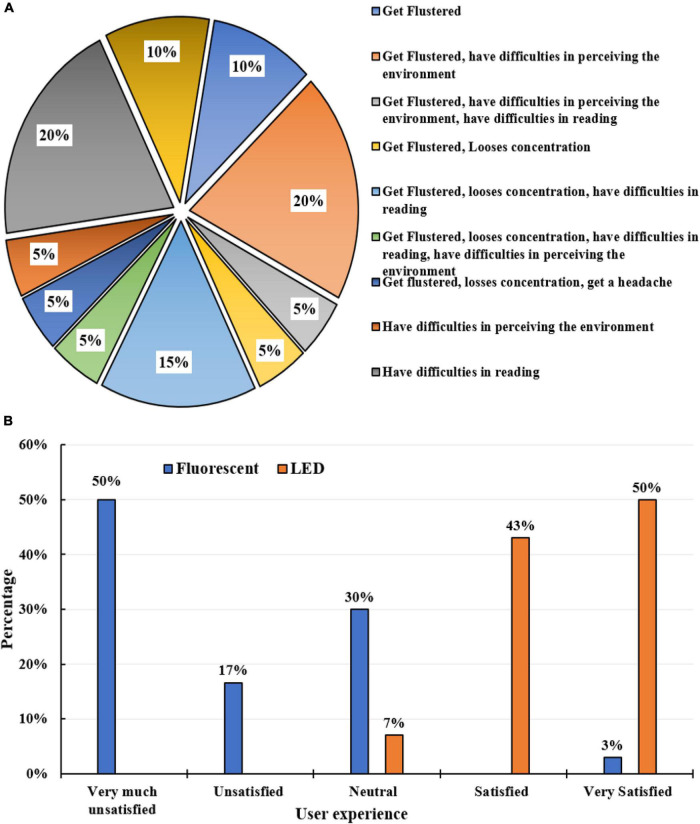
**(A)** The behavior of autistic children under low light conditions, **(B)** preferable light bulb for autistic children.

**FIGURE 4:**
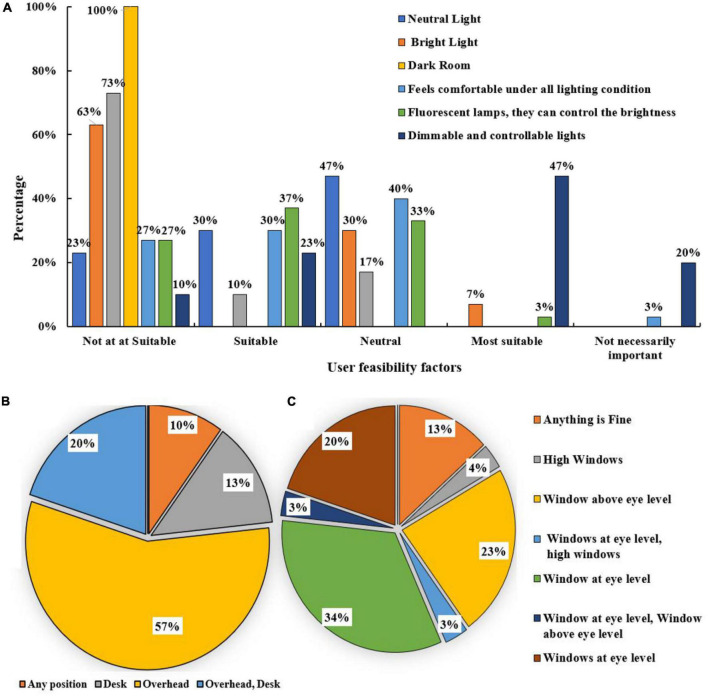
**(A)** Feasibility of autistic children under various lighting conditions, **(B)** preferable position of the light, **(C)** preferable position of window placement.

Figures 2–4 reveal that, in most cases, autistic children did not prefer to have too harsh lights, fluorescent tubes, bright lights, below-eye-level window positions, flickering lights, dark lights, and dark spaces. Children felt flustered in low light conditions and had difficulty perceiving the environment in most cases. Flickering fluorescent lights were to be avoided as they were a stress-producing factor that made the children feel agitated and uncomfortable. Autistic children are extremely sensitive to the sub-visible flicker of direct fluorescent lighting, which can hurt their eyes and cause headaches. Ideally, all lights must be easy to use and come with a control switch so that children can alter the intensity of lighting fixtures, and dim or brighten them, to suit their visual needs. Neutral lighting can calm and soothe children by fostering a relaxing environment. Ideally, lights must be task-specific and based on the circumstances and conditions of the indoor space. Proper lighting design is essential as people with ASD have a heightened response to sensory inputs. According to the survey, direct lighting should be present in rooms, but intense light or glare must be avoided, and natural daylight is preferable in as many places as possible.

Sunscreen can be provided as it can be a convenient option for autistic children who want to block out direct light diffusion. The communication and behavior of the children were observed when they were studying under low light conditions to determine if they were calm and focused or agitated and irritable to identify an autism-friendly lighting arrangement. In most of the cases, it was observed from the survey that the children had difficulty with language activities that involved reading. They struggled with words and quickly got flustered. Autistic children have characteristics with the visual perception of the environment, tend to lose concentration, and have learning difficulties. A combination of factors considered in the study may be needed to better understand their behavior within an enclosed space. This is because autism is a complex spectrum disorder with a broad range of conditions and features that differ vastly from person to person.

### Analysis of colors

**FIGURE 5:**
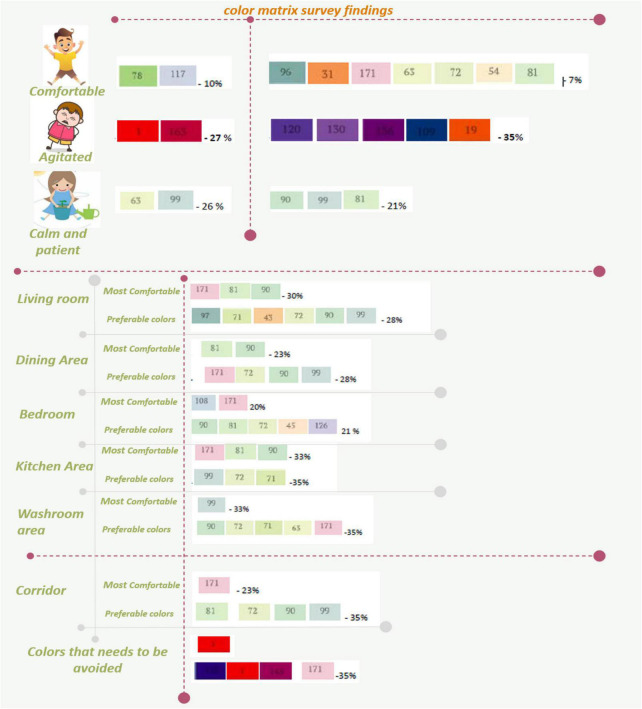
Illustration of color analysis from the survey done by the color matrix.

When conducting the survey, it was observed that most parents wanted a designated sensory space to provide an immersive sensory experience for their children. A sensory space is a specially designed and personalized therapeutic area with many sensory-friendly objects that autistic children are familiar with, which can be explored with the senses and are in different color palettes and textures. This space is designed to provide a calm and relaxing area for autistic children with sensory processing challenges to slowly habituate themselves to the visual and tactile environment. The survey reveals that it is critical to consider visual aspects at the preliminary design stage to ensure a good balance of visual elements and features in the built environment to make it a safe, secure, accessible, appealing, and comfortable for autistic children. Figure 5 illustrates the color analysis of the survey done using the color matrix.

A color-related questionnaire is handed out to the participants to determine color preferences; it is found that the most suitable colors are pastel, dull, neutral, and muted shades that are not distracting but have a tranquil effect. The chosen color scheme must accommodate visual sensitivity is of prime consideration for autistic children with sensory processing disorder as it can affect their mood, learning ability, and function. The darker shades are deemed to be unsuitable. The least preferred colors cause extreme behavioral changes like agitation, irritability, confusion, distress, anger, and aggressiveness.

The interaction of light and color is observed in this survey as their physiological effects can positively impact an autistic child. The variables defined in the table for studying the impact of light create an understanding of whether artificial lights, LED, dimmable, and incandescent bulbs are preferable depending on their effect on the sensitive eyes of autistic children (74–80). The most preferred light position is overhead, as there is no direct eye-level visibility to any artificial lights. Light visibility can be minimal to the user if the lights are flushed in the ceiling or when a false ceiling is provided. The relation of light with color and vice-versa is critical. They do not work in isolation; they work together. Both these factors combine to play an essential role in the built environment. Social contexts and family care are also essential to better servicing autistic children (81–87). Ensuring illumination comfort for autistic children by designing spaces that consider their light sensitivity is essential. The surveys conducted on autistic users offer an exceptional understanding of how they perceive color and react to it with their unusual sensory processing. This study contributes to identifying the initial set of design parameters that must be considered in an indoor environment to meet the needs of autistic children.

## Discussion

### Impact of lights

On examination of the various lighting conditions, a dark room, darker lights, and more brightness are not preferred. Natural daylight is perceived to be the best lighting for indoor spaces. Fluorescent lamps are suitable if they have the option for the brightness levels to be controlled. Neutral-colored lights are the preferred option. Most participants prefer LED lights over fluorescent lights as the harsh light from the latter tends to cause agitated behavior and extreme distress in autistic children with light sensitivity.

Another component of lighting conditions within enclosed spaces must be considered the light’s and window’s positions. Autistic children can be oversensitive or under-sensitive to bright lights, which can debilitate their functioning; strategic light and window placements can help minimize the light intensity and provide a calm and relaxed work or play environment. The overhead lights option is preferable to avoid direct light visibility, and desk lights are another option. It is observed that in most cases, the window is situated at or directly above eye level. Windows at higher levels are not preferred. The study also included the color and temperature of lighting applications while determining the light efficacy factor as they tend to psychologically impact all humans, particularly autistic children with heightened sensory sensitivity. This becomes important as there is a definite connection between lighting and behavior, as evident from behavioral changes noticed in autistic children who become aggressive and flustered if they are not comfortable with the illumination provided by the lighting arrangements. Subtle manipulation of lighting can alter the mood, behavior, and perception of autistic children. Light color, source, quality, direction, and intensity are aspects to be considered in providing an optimally lighted environment that considers the light sensitivity of its inhabitants.

### Impacts of colors

A questionnaire survey method investigates the visual aspects that significantly influence a space. According to the study, it is noted that children have a broad range of color preferences and respond distinctively and differently to various shades showing the importance of using an ASD-friendly color palette. Light, color, and space are essential components of any indoor environment that must be considered when designing for children on the autism spectrum. Architecture, as a profession, uses best design practices to devise customized environments which are engaging spaces that fulfill the specific needs of individuals, help them cope with their surroundings, encourage their independence, and positively impact their performance and behavior. In this regard, with greater recognition of effective space design’s impact on individuals with autism, designers and clinicians can consider indoor environmental aspects at the primary level and research the same issue with other interlinked factors. This study is one kind to facilitate a macroenvironment that can promote clinical services for autistic children. These results help determine the external factors that could enhance the abilities and functioning of autistic children in the built environment.

According to the survey results, light and color are significant variables within a built environment that greatly influence autistic children as they have a sensory processing disorder. Among the selected variables, light and color strongly correlate with the behavior of ASD children when compared to space and wayfinding. The results of the present study are on par with the literature (88–93). Every autistic individual is unique; each person reacts differently to the environment and sensory stimuli besides having individual preferences and aversions. As per the study, they have many color preferences and respond differently to different colors. Colors and light can be effectively deployed to bring about some desired positivity and social and behavioral change. The light sensitivity factor deals with the difficulty of sensory overload and requires further research on light rendering and acoustics. Such architectural aspects of the built environment also favor dealing with the characteristics of autistic children in addition to other clinical and non-clinical methods and techniques.

### Limitations of this case study

The number of participants involved in the present study produces substantial input to the architects and clinicians. However, further studies with all stakeholders with more populations and their interactions are necessitated to improve the built environment designs more refined for autistic children. This study is mainly focusing on the non-clinical survey approach. However, the clinical and non-clinical approaches, like measuring heart rate, speech, language skills, emotions, social interactions, other motor, and non-motor skills could produce more appropriate solutions. Further clinical studies are required to expand the demography to improve autistic-friendly built environments and designs to assist the efficiency of the support and services to autistic children. The present study’s results are mainly pertinent to design an autistic-friendly built environment on a non-clinical approach using a questionnaire survey. Thus, further non-clinical and clinical studies on several micro factors are required to expand this survey to autistic adults.

## Conclusion

The effects of light and color on the behavior of autistic children were investigated using a questionnaire survey. The full involvement of all the users was critical from the commencement to the completion of the survey. The study shows that dialogues were substantially reshaped during the color matrix questionnaire. The findings were obtained through cost-effective non-clinical methods. This was crucial from the start, as most research studies on ASD offer unclear and hypothetical results. Therapists, parents, and children actively participated in the survey sessions and offered valuable insights to designers and clinicians. The therapists and parents are vital users closely involved in meeting the needs and demands of the children under their care. Their experiences with the children offer essential data that helps understand the likes and dislikes of autistic children in the built environment regarding the lights and colors. The major conclusions of the present study are as follows.

• The standard deviation of the survey method is 2.3. The margin of error and coefficient of variation at a 95% confidence level is ± 0.85 and 0.27, respectively. This confirms that the results are reasonably within acceptable limits.
• The literary assessment and survey were utilized to comprehend the effect of colors and light in any indoor environment for children with autism.
• The lights and colors of indoor environments are the most influencing factors in promoting the harmony of autistic children compared to space and wayfinding.
• Direct lighting and natural daylighting are preferable to intense light or glare in as many places as possible.
• The spaces designed for people with ASD must address how these people perceive the environment and react to space features.
• The study proves that dull and pastel colors and muted lights are more suitable for visually sensitive autistic children.
• Lower sound levels of below 50 dB are autistic-friendly, and beyond 60 dB develops inappropriate behaviors.

Light, color, and space are preliminary considerations of any inhabited environment. There is an increased need to ensure the suitability of light and color provisions in all interior spaces intended for autistic children to make them sensory-friendly. These findings emphasize the need to understand the different parameters associated with autistic users and the benefits of designing an autistic-friendly environment.

Designers should look upon these three variables effectively for comprehensive design and further studies. By recognizing the influence of the sensory environment on autistic behavior, designers should consider these critical variables and adopt a sensory-sensitive approach to design inclusive spaces with optimum comfort. The current findings emphasize the need to understand the different sensory issues and parameters associated with autistic users and the importance of design considerations required for setting up an accommodative, supportive, neurodiverse, and autistic-friendly environment tailored to their need for positive sensory experiences. Thus, the present analysis and the scope for further research studies could benefit autistic individuals by appropriately analyzing the selective blended clinical and non-clinical techniques.

## Data availability statement

The original contributions presented in this study are included in the article/supplementary material, further inquiries can be directed to the corresponding authors.

## Ethics statement

Ethical review and approval was not required for the study on human participants in accordance with the local legislation and institutional requirements. The patients/participants provided their written informed consent to participate in this study.

## Author contributions

AN, RP, and PR contributed to the study’s conceptualization, design, and investigation. CP, RS, and SD critically verified the data and analyzed. KL, XW, and XZ contributed to the data analysis and revision of subsequent versions. All authors contributed to the article and approved the submitted version.

## Appendix A

### Appendix-1 Questionnaire

#### • Section A

• What is the age of child?
  • Which city is the child and family living?
  • What is the gender of the child?

#### • Section B

• Impact of light on autistic children.
• **Parameter 1.0**
  • How suitable is the following artificial lighting conditions for your ward? [Fluorescent tubes].
  • How suitable is the following artificial lighting conditions for your ward? [Normal bulbs].
  • How suitable is the following artificial lighting conditions for your ward? [The light is not bright].
  • How suitable is the following artificial lighting conditions for your ward? [The light is bright].
  • How suitable is the following artificial lighting conditions for your ward? [The light does not flicker].
  • How suitable is the following artificial lighting conditions for your ward? [They can dim the light by themselves (brighter/darker)].
  • How suitable is the following artificial lighting conditions for your ward? [Generally, they do not like artificial lighting].
• **Parameter 2.0**
  • How suitable is the following natural daylight conditions for your ward? [There is No direct light into the room].
  • How suitable is the following natural daylight conditions for your ward? [The light is not bright].
  • How suitable is the following natural daylight conditions for your ward? [The light is bright].
  • How suitable is the following natural daylight conditions for your ward? [They have the possibility to close the sun screening device].
  • How suitable is the following natural daylight conditions for your ward? [Generally, do not like daylighting].
• **Parameter 3.0**
  • Select the options that describe how your ward behaves when exposed to low light conditions.
• **Parameter 4.0**
  • How suitable is the following lighting conditions for your ward? [Neutral Light].
  • How suitable is the following lighting conditions for your ward? [Much of bright light].
  • How suitable is the following lighting conditions for your ward? [Preferably a little bit darker light].
• **Parameter 5.0**
  • How suitable is the following lighting conditions for your ward? [Totally dark room].
• **Parameter 6.0**
  • How suitable is the following lighting conditions for your ward? [Feels comfortable under all lighting conditions].
• **Parameter 7.0**
  • How suitable is the following lighting conditions for your ward? [Fluorescent lamps are also ok, provided they can control the brightness].
  • How suitable is the following lighting conditions for your ward? [Would make all the lights in the house dimmable and controllable].
  • Choose the preferred window option for your ward from the list below.
• **Parameter 8.0**
  • How satisfied is your ward with the following light bulb? [Fluorescent].
• **Parameter 9.0**
  • How satisfied is your ward with the following light bulb? [LED].
• **Parameter 10**
  • Choose the position of the light in any room that your ward prefers.

#### • Section C

• Choose three (color numbers; for example, 71, 107, 98) from the given color matrix that makes your ward feel comfortable.
• Choose three (color numbers) from the color matrix that makes your ward feel uncomfortable.
• Choose three (color numbers) from the color matrix that causes your ward to become very agitated.
• Choose three (color numbers) from the color matrix representing your ward’s calm and patience.
• Choose three (color numbers) for the bedroom space from the color matrix above.
• Choose three (color numbers) for the living room from the color matrix above.
• Choose three (color numbers) for the kitchen area from the color matrix above.
• Choose three (color numbers) for the dining area from the color matrix above.
• Choose three (color numbers) for the washroom area from the color matrix above.
• Choose three (color numbers) for the corridor area from the color matrix above.
• Choose three (color numbers) from the color matrix figure given that should be avoided for your ward.
• Is there a need for sensory space at home for autistic child?
• Do you feel that the visual aspect is critical for autistic children?
• Are any specific changes you made to make your ward feel safe and comfortable in your home?
• Any ideas on what you’d like to include when designing a space for an autistic child?
